# Clinical characteristics and outcome of genitourinary tuberculosis in Sri Lanka: an observational study

**DOI:** 10.1186/s12879-021-06990-z

**Published:** 2021-12-27

**Authors:** Umesh Jayarajah, Milan Gunawardene, Munipriya Willaraarachchi, Shirani Chandrasiri, Perumal Udayakumaran, Cherine Sosai, Anuruddha Abeygunasekera

**Affiliations:** 1 Department of Urology, Colombo South Teaching Hospital, No: B229 Hospital Road, Dehiwala, 10350 Sri Lanka; 2 Department of Microbiology, Colombo South Teaching Hospital, Dehiwala, Sri Lanka; 3 Department of Radiology, Colombo South Teaching Hospital, Dehiwala, Sri Lanka; 4 Department of Pathology, Colombo South Teaching Hospital, Dehiwala, Sri Lanka

**Keywords:** Genitourinary tuberculosis, TB, Extra-pulmonary TB, Sri Lanka, Observational study

## Abstract

**Background:**

Although genitourinary Tuberculosis (GUTB) is the second commonest source of extrapulmonary TB in most countries, the reported rate of GUTB in Sri Lanka remains low. The characteristics of GUTB in Sri Lanka have not been studied and documented so far. We aimed to study the clinical and imaging characteristics, treatment modalities and outcome of GUTB in Sri Lanka.

**Methods:**

Data collected from patients treated by a single urological surgeon in two institutes consecutively over a period of 21 years were analysed. All patients with a microbiological and/or histopathological diagnosis of GUTB were included. Median duration of follow-up was 24 months (range: 6–96).

**Results:**

There were 82 patients and 45 (54.9%) were men. The median age was 51 (range: 26–75) years. Most patients (39%, n = 32) had vague non-specific symptoms at presentation. Common specific symptoms at presentation were haematuria (15.8%, n = 13) and scrotal manifestations (15.8%, n = 13). Mantoux test was done in 70 patients and was > 10 mm in 62 (88.5%). Erythrocyte sedimentation rate was available in 69 patients and was > 30 mm in 54 (78.3%) patients. Chest x-ray and x-ray kidney-ureter-bladder (KUB) abnormalities were detected in 9 (11%) and 6 (7.3%) respectively. CT-urography was performed in 72 patients and abnormalities were detected in 57 (79%) patients. Forty-two patients underwent cystoscopy and 73.8% (n = 31) had abnormal findings. Microbiological diagnosis was feasible in 43 (52.4%) and rest were diagnosed histopathologically. Commonest organs involved were kidney (64.6%, n = 53), ureter (51.2%, n = 42), bladder (43.9%, n = 36) and testis/epididymis (15.8%, n = 13). One patient had TB of the prostate. All were treated primarily with anti-TB drugs however, 50 (61%) required ancillary therapeutic interventions. The majority of interventions were reconstructive surgeries (n = 20, 24.4%) followed by excisional surgeries (n = 19, 23.2%) and drainage procedures (n = 11, 13.4%). Seven patients developed serious adverse reactions to anti-TB drugs. Five patients developed a thimble bladder with disabling storage symptoms. Eight patients had deranged renal functions at diagnosis and three patients developed progressive deterioration of renal function and two patients died of end stage renal disease.

**Conclusions:**

The combination of urine for acid-fast bacilli, Mantoux test, CT-Urography, cystoscopy and histopathology is necessary to diagnose GUTB in resource-poor settings. Most ureteric strictures, non-functioning kidneys and epididymal masses need surgical treatment. Long-term follow up is essential to detect progressive deterioration of renal function.

## Background

Tuberculosis (TB) is a major global health problem especially in the developing world [1]. In 2020, TB was the thirteenth leading cause of death. Approximately 1.5 million including 214,000 people with HIV died of TB in 2020 [1]. According to the World Health Organization estimates, the incidence of TB is 10 million cases per year and 98% of them are from the developing world. Extrapulmonary TB accounts for 15–20% of reported cases [2, 3]. Among extrapulmonary TB, Genitourinary tuberculosis (GUTB) is the second commonest (20 to 40%) in most developed countries and the third commonest in most developing countries [2]. Moreover, GUTB is seen to coexist in 2–20% of patients with pulmonary TB [3]. Miliary TB with disseminated haematogenous spread to the genitourinary system is believed to be the cause for GUTB in 25–60% of patients [2]. Haematogenous seeding of primary TB infection to the kidney leads to granuloma formation which may subsequently caseate and rupture into the tubular lumen spreading to the distal urinary tract. The chronic and insidious nature of TB results in gradual damage to the genitourinary tract by a combination of chronic inflammation, necrosis, abscess formation and scarring leading to direct injury or indirect injury secondary to obstruction [2, 3].

Sri Lanka is a South Asian tropical country with a population of 22 million. Although GUTB is the second commonest source of extrapulmonary TB in most countries, the reported rate of GUTB in Sri Lanka remains very low [4]. According to the National Programme for TB control and chest diseases (NPTCCD), only 4 GUTB cases were reported out of 1966 cases of extrapulmonary TB in 2007. Furthermore in 2019, only 59 GUTB cases were reported out of 2431 cases of extrapulmonary TB (8900 TB cases in total) [5]. This may be due to failure in case detection or deficiencies in proper reporting and documentation. In Sri Lanka, the characteristics of GUTB are not known due to paucity of data [4]. Only few case reports have been published describing rare manifestations of GUTB [6–9]. Knowing the disease characteristics is vital to establish early diagnosis, treatment and prevention of chronic kidney disease. Therefore, we aimed to study the clinical and imaging characteristics, diagnostic features, treatment modalities and outcome of GUTB in Sri Lanka.

### Methods

An observational study was conducted at two tertiary care Urology Units in Sri Lanka, first in Karapitiya Teaching Hospital, Galle from 1.1.2000 to 30.11.2009 and subsequently at Colombo South Teaching Hospital, Dehiwala from 1.12.2009 to 30.6.2021 (a total period of 21 years) where the senior author worked. All patients with a diagnosis of GUTB, i.e. TB involving the kidney, ureters, bladder, urethra, prostate, testis and epididymis were included in the analysis. All study participants were referred to the Urology Clinic as routine referrals or admitted directly to the emergency department with acute clinical presentations. None of the patients were referred with a diagnosis of GUTB. The data were collected prospectively from the patients at the point of diagnosis and the records were updated during their routine follow up clinic visits. Since the data collected were information that was gathered during routine clinical care, there was no special interview done specifically for the study. Thus, this was a retrospective analysis of prospectively collected data. Approval for the study was obtained from the Ethics Review Committee of the Colombo South Teaching Hospital. The study was performed in accordance with the Declaration of Helsinki and all methods were performed in accordance with the relevant guidelines and regulations.

Clinical characteristics including comorbidities, contact or past history of TB and clinical symptoms and signs were recorded. All patients underwent basic biochemical assessment including erythrocyte sedimentation rate (ESR) and serum creatinine. Mantoux test was done in all patients except when the purified protein derivative was not available for the skin test. An induration of ≥ 10 mm was recorded as a positive test in non-immunocompromised patients [3]. Ultrasound scan of the kidney, ureter, bladder and prostate (US-KUBP) and X-ray KUB were performed in all patients. Patients with symptoms related to the scrotum underwent an additional scrotal ultrasonography. Computed tomography urogram (CTU) was performed in all patients except in those with isolated scrotal symptoms and signs and negative US-KUBP and X-ray KUB. Abnormal findings noted in imaging were denoted according to the anatomical region and involved organ.

Investigations such as urine for acid fast bacilli (AFB), urine for TB culture and polymerase chain reaction (PCR) were performed in order to obtain a microbiological diagnosis. However, in a subset of patients, some of these investigations were not performed due to non-availability of reagents and resources. Selected patients underwent a cystoscopy and bladder biopsy. Indications for cystoscopy included history of haematuria, lower urinary tract symptoms or ultrasonographic or CTU evidence of bladder abnormalities such as increased bladder wall thickness, contracted bladder and mass lesions in the bladder. Bladder biopsy was performed in those with evidence of contracted bladder, mass lesion or inflammatory changes of the urothelium. A histological diagnosis GUTB was made when aggregates of epithelioid histiocytes, Langhans giant cells and caseating necrosis were present [10].

All patients were screened for pulmonary TB with a clinical assessment and mandatory chest X-ray and sputum AFB. TB of other regions were screened with a clinical history and examination of the lymph nodes and spine. Those with positive findings were subjected to further evaluation.

A diagnosis of GUTB was made in the presence of at least one of the following criteria in addition to clinical features: (1) at least one out of three urine samples were positive for AFB using Ehrlich–Ziehl–Neelsen (EZN) technique. Three consecutive, early morning, mid-stream samples of urine were obtained for the test (2) positive urine or tissue culture for *Mycobacterium tuberculosis* complex (3) positive PCR for *Mycobacterium tuberculosis* complex (4) histological evidence of TB such as chronic granulomatous inflammation with Langhans giant cells with or without caseation [3]. The reporting of histopathology and radiological imaging were done partly by authors of the study. However every patient was discussed at a multidisciplinary team (MDT) meeting where many specialists on the subject who were not authors of the study were involved in final reporting and decision making.

Patients were commenced on anti-TB therapy (ATT) as direct observed therapy (DOT) which is mandatory in Sri Lanka to enhance compliance and minimise default rate and lost to follow up. The adverse effects of ATT such as skin reactions and hepatotoxicity were also recorded. Hepatotoxicity due to ATT was defined as (1) at least a fivefold elevation in alanine transaminase (ALT) and/or aspartate transaminase above the upper limit of normal (2) more than 1.5 mg/dL increase in the total bilirubin level (3) clinical features of acute liver derangement such as loss of appetite, jaundice, vomiting, nausea, encephalopathy and at least a threefold rise in ALT and/or AST levels [3].

Selected patients not responding to ATT or who developed complications such as stricture or abscess formation underwent invasive procedures. These included minimally invasive procedures such as percutaneous drainage, cystoscopy and stenting and open procedures such as open drainage and reconstructive surgical procedures. Complications such as thimble bladder syndrome and progressive deterioration of renal function were also recorded. Estimated GFR (eGFR) was used to measure renal function. Compared to the eGFR at diagnosis, an increase of eGFR ≥ 10% at completion of treatment was defined as “recovery”. An increase or decrease of 0–9% was defined as “stability” and a lowering of ≥ 10% was considered as “deterioration” [3]. End stage renal disease (ESRD) was defined as eGFR less than 15 mL/min/1.73 m^2^ or requiring renal replacement therapy or transplant [11]. Thimble bladder syndrome was defined as disabling storage symptoms with bladder capacity of < 150 mL as measured by filling the bladder with saline during cystoscopy.

All patients were seen 6 months after completion of ATT with US-KUBP and serum creatinine. Thereafter, those with structural abnormalities of the urinary tract in US-KUBP, high serum creatinine, single functioning kidney and those who had reconstructive surgery were followed up with annual US-KUBP and serum creatinine.

Statistical analyses were performed using SPSS software version 17. Data were expressed as frequency and percentages or median and range as relevant. Non-parametric tests were used to determine associations and statistical significance. Associations between categorical variables were determined using Chi-square test and associations between continuous and categorical variables were determined using Mann–Whitney U test. A p value of less than 0.05 was considered statistically significant.

## Results

During the study period of 21 years, a total of 120 cases were suspected to have GUTB. However, based on the diagnostic criteria, 38 cases were excluded and a total of 82 patients were included in the analysis.

### Clinical characteristics and basic investigations

The majority were males (54.9%, n = 45). The median age of the sample was 51 years (range: 26–75). The basic demography, predominant clinical presentation and investigations are summarised in Tables 1 and 2. Most patients (39%, n = 32) had vague non-specific symptoms at presentation. Common specific symptoms at presentation were haematuria (15.8%, n = 13) and scrotal manifestations (15.8%, n = 13) (Table 1). Past history of TB or contact history of TB was seen in only 2 (2.4%) patients.

**Table 1:** Basic demographic and clinical characteristics

	N	%
Age category (years)		
21–30	4	4.9%
31–40	17	20.7%
41–50	19	23.2%
51–60	23	28.0%
61–70	16	19.5%
71–80	3	3.7%
Gender		
Male	45	54.9%
Female	37	45.1%
Predominant complaint		
Non-specific vague symptoms. e.g. abdominal discomfort, malaise, mild dysuria	32	39.0%
Haematuria	13	15.9%
Scrotal mass	12	14.6%
Colicky abdominal pain	7	8.5%
Symptoms of septicaemia	5	6.1%
Lower urinary tract symptoms	5	6.1%
Persistent severe dysuria	5	6.1%
Scrotal sinus	1	1.2%
Faecaluria	1	1.2%
Dyspareunia	1	1.2%
Examination findings		
Nodular prostate	3	3.7%
Scrotal mass	12	14.6%
Scrotal sinus	1	1.2%
Abdominal wall abscess	1	1.2%

**Table 2:** Summary of basic biochemical investigations and X-ray findings

	N/mean*	% (Range)
Erythrocyte sedimentation rate (mm/h)	57*	(6–140)
Mantoux		
Strongly positive (≥ 15 mm)	33	47.1%
Positive (10–14 mm)	29	28.5%
Equivocal (5–9 mm)	8	9.8%
Negative	0	0.0%
Not available	12	14.6%
Serum creatinine (mg/dL	3.08*	(1.60–5.83)
eGFR (mL/min/1.73 m^2^)	22.13*	(10.0–45.0)
Chest X-ray		
Fibrosis	2	2.4%
Opacities	6	7.3%
Effusion	1	1.2%
Normal	73	89.0%
X-ray KUB		
Calcifications	5	6.1%
Collapsed L1 vertebra	1	1.2%
Normal	76	92.7%

Asterisk “*” indicates mean values

Mantoux was performed in 70 patients and all were either positive (> 10 mm) (n = 62, 88.6%) or equivocal (> 5 mm) (n = 8, 11.4%) (Table 2). Only 18.2% of scrotal TB had strongly positive Mantoux compared to 52.5% of others having a strongly positive result (p = 0.036). ESR was available in 69 patients and was > 30 and > 50 in 54 (78.3%) and 33 (47.8%) patients respectively. The mean ESR was significantly lower in those with scrotal TB than others with urinary tract involvement (27 vs. 61, p = 0.002). Only 8 (9.8%) patients had elevated serum creatinine (> 1.5 mg/dL) at the time of diagnosis. Chest x-ray and x-ray KUB abnormalities were detected in 9 (11%) and 6 (7.3%) respectively (Table 2).

### CT-Urogram

CT-urogram was performed in 72 (87.8%) and abnormalities were detected in 57 (79.2%) patients. These included ureteric strictures (n = 19), renal inflammation or abscess (n = 9), non-functioning kidneys (n = 6), stones or calcifications (n = 6), contracted bladders (n = 5) and masses (n = 4) (Table 3).

**Table 3:** Summary of CT-urogram findings at the time of diagnosis

CT-Urogram findings	Total (N = 72)		Male (N = 35)		Female (N = 37)	
	N	%	N	%	N	%
Presence of bilateral abnormalities	9	12.5%	7	20.0%	2	5.4%
Presence of upper and lower tract anomalies	3	4.2%	3	8.6%	0	0.0%
Presence of isolated lower tract anomalies	3	4.2%	1	2.9%	2	5.4%
Presence of isolated upper tract anomalies	49	68.1%	21	60.0%	28	75.7%
Ureteric stricture	18	25.0%	7	20.0%	11	29.7%
Renal stones	3	4.2%	2	5.7%	1	2.7%
Renal or ureteric calcification	3	4.2%	2	5.7%	1	2.7%
Small bladder	3	4.2%	3	8.6%	0	0.0%
Renal mass	3	4.2%	1	2.9%	2	5.4%
Bladder mass	1	1.4%	0	0.0%	1	2.7%
Non-functioning kidney	6	8.3%	2	5.7%	4	10.8%
Renal abscess	4	5.6%	2	5.7%	2	5.4%
Peri-renal abscess	2	2.8%	1	2.9%	1	2.7%
Spinal TB	2	2.8%	2	5.7%	0	0.0%
Psoas abscess	3	4.2%	2	5.7%	1	2.7%

### Cystoscopy

Out of 42 patients who underwent cystoscopy, 31 (73.8%) had abnormal findings necessitating a bladder biopsy. Commonest abnormality was inflammatory changes in the bladder which was seen among 30 (71.4%) patients. Other anomalies included mass lesions of the bladder (n = 2), contracted bladder (n = 2) and golf hole ureteric orifice (n = 1). The histopathology was suggestive of TB in 25/31 patients (80.6%). All positive bladder biopsies had severely inflamed urothelium of the bladder.

### Diagnosis

Microbiological diagnosis was achieved in 43 (52.4%) patients, while the rest were diagnosed by histopathological features. The mode of obtaining the specimen for histopathological evaluation included cystoscopic bladder biopsy (n = 25, 30.5%), and/or open surgical biopsy or assessment of resected specimen [testis: n = 13 (15.8%); ureter: n = 17 (20.7%); kidney: n=7 (8.5%); colovesical fistula: n = 1 (1.2%)]. Only 6 patients showed caseation in histopathology. Commonest organs involved were kidney (64.6%, n = 53), ureters (51.2%, n = 42), bladder (43.9%, n = 36) and testis/epididymis (15.8%, n = 13). Only one patient had prostatic TB and none had urethral TB.

### Pulmonary and other extra-pulmonary manifestations

Four patients had concurrent pulmonary TB and other areas involved were psoas abscess (n = 3), spine (n = 2), anterior abdominal wall (n = 1) and colon (n = 1).

### Treatment and follow up

All were treated primarily with anti-TB therapy; however, 50 (61%) patients had required ancillary invasive interventions. Median duration of follow-up was 24 months (range: 6–96). The preferred initial treatment was 2 months of four drugs, followed by 4 months of maintenance with isoniazid and rifampicin. The interventions performed were reconstructive surgeries (n = 20, 24.4%), excisional surgeries (n = 19, 23.2%) and drainage procedures (n = 11, 13.4%). A summary of therapeutic interventions and their indications are given in Table 4. High ESR (> 50) or positive Mantoux were not predictive of the need for therapeutic interventions (p = 0.126 and p = 0.744, respectively).

**Table 4:** Summary of therapeutic interventions and their indications

Type of intervention	N	Indications
Ureteric reimplantation	14	Lower ureteric strictures (n = 14)
Boari flap reconstruction	2	Lower and mid ureteric stricture (n = 1) Lower ureteric stricture (n = 1)
Ureteroureterostomy	1	Upper ureteric stricture (n = 1)
JJ stenting (via cystoscopy)	8	Lower ureteric stricture (n = 3) Mild ureteric stenosis (n = 2) Lower ureteric stricture with ureteritis cystica (n = 1) Psoas abscess with hydronephrosis and hydroureter (n = 1) Pyonephrosis (n = 1)
Excision of mass	4	Epididymal mass (n = 4)
Epididymectomy	4	Epididymal mass (n = 4)
Orchidectomy	4	Large testicular/epididymal mass (n = 4)
Nephrectomy	5	Non-functioning kidney (n = 5)
Nephroureterectomy	2	Non-functioning kidney with a ureteric mass (n = 1) Extensive nephroureteric calcification (n = 1)
Percutaneous nephrostomy	1	Pyonephrosis (n = 1)
Guided drainage	1	Renal abscess (n=1)
Open drainage (twice)	1	Psoas, peri-renal and anterior abdominal wall abscesses (n = 1)
Resection of colovesical fistula tract and repair	1	Colovesical fistula (n = 1)
Patient refused ureteric reimplantation	2	Lower ureteric stricture (n = 2)

### Drug toxicity

Seven patients (5 were women) developed significant drug toxicity. Six patients developed hepatitis with increased liver enzymes. All had discontinuation of isoniazid and rifampicin. Four patients had improvement of liver enzymes (ALT less than 50 U/L in 2–3 weeks) after discontinuation of therapy. Isoniazid and rifampicin were restarted and increased gradually until the optimum dose was achieved without subsequent complications. In one patient, streptomycin, moxifloxacin and ethambutol were given during the first 3 weeks (after stopping isoniazid and rifampicin) due to clinical evidence of severe disease. One patient died of progressive liver derangement and liver failure in 3 weeks.

One patient developed extensive skin rash and itching and had low blood pressure without any evidence of hepatotoxicity. The patient was treated with prednisolone and chlorpheniramine. Desensitization was performed successfully with gradual increment of drugs after 2 weeks while continuing prednisolone.

### Renal impairment

Eight patients had serum creatinine more than 1.5 mg/dL at the time of diagnosis (median: 2.4 mg/dL; range: 1.6–5.83). One patient had normal serum creatinine at diagnosis that gradually rose to 3.2 mg/dL after 4 years. He is currently on an indwelling catheter for thimble bladder syndrome. Of the 8 patients with deranged serum creatinine at diagnosis, three had progressive deterioration of renal function and two of them (one had thimble bladder) died of end stage renal disease while receiving renal replacement therapy in the form of haemodialysis (3 years and 5 years after starting ATT). One underwent a kidney transplant. Recovery of renal functions and stable renal functions were seen in three and two patients respectively.

### Thimble bladder syndrome

Five patients (2 males and 3 females) had severe storage symptoms and bladder capacity less than 150 mL. Of the two males, one died of progressive renal impairment and ESRD and the other patient is on follow up with a serum creatinine of 3.2 mg/dL. Both needed indwelling catheters to manage their disabling storage symptoms. The three women were managed without catheters. All refused bladder augmentation because of reluctance to perform clean intermittent catheterisation post-surgery. Additionally, all patients received antimuscarinic drugs such as oxybutynin or tolterodine.

### Relapses

Two patients with relapsing disease were detected. One patient who was treated for renal TB presented with an epididymal mass and sinus formation after 4 years of treatment completion. He was treated with the Category two regime which comprised of the usual ATT and streptomycin. Another patient who underwent nephrectomy for renal TB presented with severe haematuria, dysuria and frequency after 2 years of treatment completion. Cystoscopy showed a severely inflamed bladder epithelium and the bladder biopsy was positive for TB. The patient was treated with ATT for 9 months. Streptomycin was avoided as he was having a single kidney.

## Discussion

### Summary of results

We report our 21-year experience in the management of GUTB in a resource limited setting. Although we suspected TB in a larger cohort, only a subset was confirmed to have TB based on the microbiological and histological criteria. Although granulomata with Langhans giant cells were seen in histology, caseation was rare. This may be due to the effect of quinolones that are used liberally for patients with urinary symptoms. All ureteric strictures were treated with stenting and prednisolone. However, most ureteral strictures needed reconstruction with ureteric reimplantation. Drug toxicity was more common among females. Few patients developed thimble bladder syndrome and progressive deterioration of renal function.

### Regional comparison of findings

In our cohort, kidney, ureter, bladder, and testis/epididymis are the common organs involved. A study from India by Bansal et al., found that among 60 cases, the commonest organ affected was the kidney (n = 34) followed by the bladder (n = 25) and the ureter (n = 20) [12]. Another retrospective study from India by Krishnamoorthy et al. showed that among 110 cases, renal, ureter and bladder involvement was seen among 70, 30 and 18 respectively similar to our study [13]. Interestingly, urethral and prostate involvement was extremely rare in our cohort. Although many urethral fistulae and masses were evaluated with biopsies and microbiological investigations for TB during the study period, none became positive for TB. Although prostate gland involvement is less common, a few incidental prostate TB cases were identified in chippings after trans-urethral resection in our neighbouring country—India [13, 14]. However, we were able to detect only one patient during the last two decades. The reason for this regional variation seems unclear. In our cohort, around 15% of GUTB presented with scrotal manifestations such as a lump or rarely a sinus. In these patients, the positivity of ESR and Mantoux were significantly lower than those with abdominal manifestations. Therefore, high degree of suspicion and obtaining a tissue sample for further evaluation is mandatory to clinch the diagnosis.

### Diagnosis of GUTB

Diagnosis of GUTB is a challenge due to the rarity of the disease and varying clinical presentation [15]. Furthermore, basic imaging such as X-rays and ultrasonography are not reliable. X-ray KUB may show ill-defined or diffuse calcifications, but this is becoming rare and in our cohort, such features were seen in 7.3% only [4]. Features in ultrasonography are also non-specific and may show calyceal dilatation, parenchymal destruction, calcification, hydronephrosis or collections [4, 16]. However, it is not useful in most patients except in those with scrotal manifestations [17]. CT-urogram was a useful investigation to detect anatomical abnormalities that are characteristic of TB such as ureteric stricture, unusual parenchymal calcification, renal/peri-renal abscesses and non-functioning kidney [4, 18, 19]. Cystoscopy and bladder biopsy was very useful in clinching the diagnosis in those with haematuria and lower urinary tract symptoms [20]. Presence of inflammation, small capacity bladder, inflammatory masses and golf hole ureteric orifices were characteristic.

It is important to note that microbiological or histopathological confirmation is mandatory to start ATT due to the rarity of the disease and the potential side effects of ATT [21, 22]. Starting ATT on clinical grounds without proper microbiological or histological confirmation should be discouraged as it may cause more harm. In our cohort, 7 patients developed serious adverse effects due to ATT and one patient died of liver failure. It is important to note that urine for AFB, Mantoux and granulomata are notorious to give rise to false positive results thus, use in isolation is not recommended by the experts [21]. Nevertheless, combination of these investigations is very useful to detect GUTB as TB culture or PCR may become negative with widespread use of quinolones. Thus a combined effort of surgeons, radiologists, pathologists, microbiologists and chest physicians are required for the diagnosis of GUTB [23].

### Treatment of GUTB

Many patients required some form of intervention in addition to ATT for 6 months. However, 6 months of ATT may not be adequate in patients with a high burden of diseased tissue [21]. Unlike in pulmonary TB, surgery in GUTB is an important adjunct [18]. Surgical procedures should be done after 4–6 weeks of anti TB therapy. Pus should be drained either through open or minimally invasive approach. Sinus formation after drainage of tuberculous abscess was not problematic and all healed with ATT. Extirpative/excision surgery such as nephrectomy or orchidectomy is required in extensive disease not responding to ATT or when there is a suspicion of malignancy [18]. A reconstructive procedure was necessary for almost all our patients with ureteric stricture as they did not respond to ATT and steroids. Although TB causes bladder fibrosis, ureteric reimplantation or Boari flap were technically feasible with satisfactory healing as opposed to bladder fibrosis after radiotherapy. Bladder augmentation procedures are required for thimble bladder with disabling storage symptoms [18]. However, all five patients refused augmentation procedure as they did not prefer clean intermittent catheterization post-surgery. Finally, complex fistulae such as colovesical fistulae would need anatomical delineation followed by excision and reconstruction [18].

Prognosis of GUTB is satisfactory in general and in most patients, there was no evidence of impaired renal function at the time of diagnosis. Even those with renal dysfunction had their serum creatinine level stabilised or recovered towards normal with ATT. However, a minority developed end-stage disease especially in those with thimble bladder and bilateral renal involvement. We had three patients with ESRD and two died while on haemodialysis and one underwent successful renal transplant. Therefore, long term follow up is essential in patients with GUTB to identify those with progressive fibrosis and renal impairment. Relapses were uncommon after successful treatment completion.

### Patterns of GUTB in Sri Lanka

We noticed several changes in the pattern of GUTB in Sri Lanka over the recent years. The number of cases is increasing in Sri Lanka as evident by the 4 cases reported in 2007 and 59 cases in 2019 [4, 5]. This may be due to increased awareness, detection and reporting. Furthermore, advancements in the facilities such as better availability of reagents for TB detection, availability of tests giving rapid results such as PCR and advanced nucleic acid replication tests—GeneXpert also would have contributed [21]. Since GeneXpert test is not recommended for urine, detection of acid-fast bacilli in urine smears was useful in our resource-limited setting [21]. Increased cases may be a reflection of increased prevalence of immunocompromising conditions such as diabetes mellitus and malignancies in the Sri Lankan population [24–26]. Multi drug resistance is becoming an emerging problem although it was not seen among our GUTB patients [21]. In Sri Lanka, an average of 16 patients with multi drug resistant TB per year has been reported during the period of 2018 to 2020 [5]. Fortunately, isolated GUTB is not contagious unlike active pulmonary TB and therefore, precautions such as social distancing are not required during the initial phase of treatment [21]. The World Health Organization (WHO) has proposed the “End TB Strategy” to eliminate TB with a target of reduction of TB mortality by 95% and incidence by 90% by 2035 [27]. To achieve this goal, a high degree of suspicion and early case detection with prompt treatment is the way forward. In Sri Lanka, directly Observed Treatment (DOT) with polypills rather than individual drugs are implemented to enhance compliance and minimise default rate [21].

### Limitations

There are several limitations in this study. This is an observational study based on patients that were diagnosed and treated by a single urological surgeon in two consecutive centres. However, we believe that our study is generalizable to most part of Sri Lanka as the urology units of the two institutions cater to a large catchment population throughout the island. Although many were referred with non-sinister symptoms, some had radiological evidence of urinary tract obstruction. This may have added some bias of picking up more severe disease; however this should have been minimal. As this is a resource limited setting, certain investigations were not carried out at times due to non-availability of facilities and reagents uninterruptedly. Certain important data like presence of comorbidities like diabetes mellitus and other risk factors has not been recorded. Follow-up data is incomplete as information from patients who attended routine follow-up visits only have been collected. Nevertheless, this is a comprehensive report of two decades of experience and the first report of GUTB from Sri Lanka.

## Conclusions

The best way to control TB is by early case detection and complete treatment. For this, a high degree of suspicion and a combination of investigations in all suspected patients is crucial. Thereafter an individualised decision based on inputs from a multi-disciplinary team is essential for the diagnosis of GUTB and there is no single easy gold standard test. Therefore, investigations such as urine for AFB, Mantoux test, CT-urography and histopathological evaluation are essential adjuncts to diagnose GUTB. Surgery is an important modality of treatment in most patients in addition to mandatory ATT. Follow up is needed after completion of ATT as some may progress to ESRD.

## Data Availability

The data used in the above analysis will be available on reasonable request from the corresponding author.

## Abbreviations

TB: Tuberculosis
GUTB: Genitourinary tuberculosis
NPTCCD: National Programme for Tuberculosis control and chest diseases
AFB: Acid fast bacilli
EZN: Ehrlich–Ziehl–Neelsen
ESR: Erythrocyte sedimentation rate
ALT: Alanine transaminase
AST: Aspartate transaminase
US: Ultrasound
KUBP: Kidney Ureter Bladder Prostate
PCR: Polymerase chain reaction
CT: Computed tomography
MDT: Multidisciplinary team
ATT: Anti-TB treatment
eGFR: Estimated glomerular filtration rate
ESRD: End-stage renal disease
SPSS: Statistical package for social sciences
DOT: Directly observed treatment
WHO: World Health Organization
